# Do assessments of cardiorespiratory and muscular fitness influence subsequent reported physical activity? A randomized controlled trial

**DOI:** 10.1186/s13102-021-00295-z

**Published:** 2021-06-15

**Authors:** James T. Langland, Neeraj Sathnur, Qi Wang, Andrew P. J. Olson

**Affiliations:** 1 Department of Medicine, Division of General Internal Medicine, University of Minnesota, 420 Delaware St SE, MMC 784, Minneapolis, MN 55455 USA; 2 Department of Medicine, Division of Cardiology, University of Minnesota, Minneapolis, MN USA; 3 Clinical/Translational Science Institute, University of Minnesota, Minneapolis, MN USA

**Keywords:** Cardiorespiratory fitness, Grip strength, Physical activity, Exercise, Exercise promotion

## Abstract

**Background:**

Regular physical activity and exercise provide many health benefits. These health benefits are mediated in large part through cardiorespiratory fitness and muscular strength. As most individuals have not had an assessment of their personal cardiorespiratory fitness or muscular strength we investigated if measurements of cardiorespiratory fitness and muscular strength would influence an individual’s subsequent self-reported exercise and physical activity.

**Methods:**

Volunteer subjects at a State Fair were randomized in 1:1 parallel fashion to control and intervention groups. The baseline Exercise Vital Sign (EVS) and type of physical activity were obtained from all subjects. The intervention group received estimated maximum oxygen uptake (VO_2_max) using a step test and muscular strength using a hand grip dynamometer along with age-specific norms for both measurements. All subjects were provided exercise recommendations. Follow up surveys were conducted at 3, 6 and 12 months regarding their EVS and physical activity.

**Results:**

One thousand three hundred fifteen individuals (656 intervention, 659 control) were randomized with 1 year follow up data obtained from 823 subjects (62.5%). Baseline mean EVS was 213 min/week. No change in EVS was found in either group at follow-up (*p* = 0.99). Subjects who were less active at baseline (EVS < 150) did show an increase in EVS (86 to 146) at 6 months (*p* < 0.05). At 3 months the intervention group increased resistance training (29.1 to 42.8%) compared to controls (26.3 to 31.4%) (*p* < 0.05). Lifestyle physical activity increased in the intervention group at 3 months (27.7 to 29.1%) and 6 months (25%) whereas it declined in the control group at 3 months (24.4 to 20.1%) and 6 months (18.7%) (*p* < 0.05).

**Conclusion:**

Providing VO_2_max estimates and grip strength did not produce an increase in overall physical activity. The EVS and exercise recommendations did however produce an increase in physical activity in less active individuals. In a very active population the VO_2_max estimate and measured grip strength did increase lifestyle activity and resistance training. Wider adoption of these measures could be effective in promoting physical activity and resistance training.

**Trial registration:**

clinicaltrials.gov NCT03518931 Registered 05/08/2018 -retrospectively registered.

## Background

Physical activity and regular exercise are important components of a healthy lifestyle. The benefits of physical activity and exercise include lower all-cause mortality [1, 2]**,** reduced cardiovascular disease (CVD) [3], improved blood pressure [4], lower triglycerides, increased High Density Lipoprotein cholesterol [5], less depression [6], less anxiety [7], improved cognitive function [8], and improved glycemic control in both type 1 [9] and type 2 diabetics [10]. Despite the benefits of a regular exercise program, many individuals do not maintain sufficient physical activity and an increasing portion of the adult population engage in no leisure time physical activity [11]. Developing techniques to help motivate individuals to be more physically active can have important public health benefits.

The benefits of exercise are mediated in large part through cardiorespiratory fitness (CRF) [12] and muscular fitness (MF) [13]. Although there is a strong genetic component to CRF it can be improved with regular exercise and physical activity [14]. It is well established that increased CRF is associated with better functional ability, improved cardiovascular health and reduced total mortality [12]. Individual measurements of CRF are a more powerful predictor of mortality than more traditional cardiovascular risk factors such as systolic blood pressure and total cholesterol [15]. CRF is best measured by maximum oxygen uptake (VO_2_max) representing one’s maximum ability to deliver and consume oxygen during activity. Despite the importance and prognostic significance of CRF and VO_2_max they are not routinely measured or estimated in the general population or the clinical setting [16]. Measuring VO_2_max typically involves a maximal exercise session which limits its availability and utility. A self-paced step test has been validated in a primary care setting as a safe and simple method of approximating VO_2_max and CRF [17].

MF and strength also mediate the beneficial effects of exercise. Increased muscular strength is strongly associated with a lower all-cause and CVD mortality [13]. The reduction in all-cause mortality associated with muscular strength has been found to be independent of CRF [18]. Increased muscular strength improves metabolic and cardiovascular risk markers [19] and reduces the risk of developing metabolic syndrome [20] . Exercise, especially resistance training, can increase muscular strength at any age [21, 22]. Although MF and strength have important metabolic and health implications they also are not routinely assessed in the general population or in the health care setting [23]. A handgrip dynamometer has been shown to be a cost-effective clinical marker of sarcopenia and correlates with lower extremity muscle power and poor mobility [24]. A large longitudinal population study found that measurement of grip strength is a simple, inexpensive risk-stratifying method for all-cause death, cardiovascular death, and CVD [25].

Given the major health implications of CRF and MF we hypothesized that providing individuals with an estimate of their CRF and VO_2_max via the step test and MF via grip strength along with educational information and normative data would be effective in motivating an overall increase in subsequent exercise and physical activity. We also secondarily hypothesized that this increase would be through increased cardio and resistance exercise.

## Methods

### Study population

The study was undertaken at the Minnesota State Fair over 12 days in late August and early September 2014 and 2015. Subjects were self-selected when they volunteered to participate while exploring the University of Minnesota’s Driven to Discover Building on the Minnesota State Fairgrounds. Exclusion criteria were age < 18 years, pregnancy, history of heart disease, syncope, chest pain, dyspnea, beta blocker or non-dihydropyridine calcium channel blockers use or evidence of any unstable medical condition. We estimated that we needed to enroll 440 individuals (220 per group) to detect an increase of 15% in EVS with 95% confidence level.

### Enrollment and testing protocol

Subjects were randomized in a parallel fashion with 1:1 allocation via sealed envelope to either the control or the intervention group. All subjects provided signed written informed consent and had self-reported weight and height recorded and heart rate and blood pressure measured. The Exercise Vital Sign (EVS) [26] was used to document current exercise activity and was recorded for all subjects. The EVS is the product of the answers to two questions: “On average, how many days per week do you engage in moderate to strenuous exercise like a brisk walk” and “On average how many minutes do you engage in exercise at this level”. We also inquired as to the types of self-reported physical activity performed (with examples provided): None, Cardio (walking, running, biking swimming), Sports (tennis, basketball, dancing etc.), Resistance Exercises (weight lifting, resistance bands etc.), Lifestyle Activities (yard work, mowing, raking, digging etc.), Balance/Flexibility Exercises (yoga, Tai chi etc.) and Other. We capped the maximum EVS at 840 by limiting reported exercise time to 120 min/day.

All subjects in both groups were provided educational information on recommended physical activity based on the American College of Sports Medicine Guidance for Prescribing Exercise [27]. Those randomized to the control group did not undergo further testing.

The intervention group had grip strength measured in the dominant arm with a J00105 hand-grip dynamometer (Lafayette Instrument). Subjects were allowed to adjust the dynamometer to their hand size, chose their most comfortable arm position and whether to stand or sit for the testing. The highest value achieved in three attempts was recorded. Subjects were given a brief demonstration of the step test procedure by study staff but no opportunity to practice. Heart rate was recorded pre and post testing and using previously published protocols [15] VO_2_max was estimated using the timed 20 step protocol at “normal” speed. No problems or side effects were encountered during any of the measurements. The measured grip strength and the calculated VO_2_max were provided to subjects along with age specific norms for both the grip strength [28] and VO_2_max [29]. In 2014 participants were given an age specific “good” norm VO_2_max and in 2015 were provided a “superior” norm for VO_2_max [29] to determine if increasing the normative value would have a greater impact on subsequent exercise behavior.

### Follow-up data collection and analysis

All study participants in both the control and intervention groups were contacted via email at 3, 6 and 12 months following enrollment to determine their current EVS and type of physical activity after which the trial was stopped and no further contacts were attempted. The change in EVS from baseline for both the intervention and control group was compared at 3, 6, and 12 months. Pre-specified subgroup analysis was also performed based on baseline physical activity levels. Participant baseline characteristics were summarized by group using descriptive statistics. Types of exercise were compared between groups using the Chi-square test. For participants who provided data at all 4 time points, the change in EVS over time were evaluated using linear mixed models. Models included fixed effect of intervention, time, and their interaction, and accounted for correlations among repeated measures. Tukey method was used to adjust for multiple comparisons. Subgroup analysis was conducted to examine change in EVS based on baseline physical activity levels. Analysis was performed using Statistical Analysis Software (version 9.3, SAS Institute Inc., Cary, NC).

## Results

### Study population and baseline measurement

Total study enrollment was 1315 subjects (776 in 2014 and 539 in 2015) with 659 subjects assigned to the control group and 656 subjects to the intervention group. Mean age was 46.0 (range 18–92), males accounted for 63.0% of the total, and 92.6% of all participants were white. Table 1 shows the demographic characteristics and baseline EVS of the control and intervention groups.

**Table 1:** Baseline demographics

	Overall	Control	Intervention
Number of subjects	1315	659	656
Age, mean (range)	46.0 (18–92)	44.9 (18–92)	47.1 (18–88.5)
Males, N (%)	829 (63.0%)	418 (63.2%)	411 (62.3%)
Whites, N (%)	1218 (92.6%)	607 (92.0%)	611 (92.6%)
BMI Kg/M^2^, mean (range)	25.8 (16.0–61.2)	26.0 (16.0–61.2)	25.6 (16.0–56.6)
EVS minutes/week, mean (SD)	213.2 (176.6)	211.7 (168.7)	214.6 (184.1)

*BM* Body mass index *EVS* Exercise Vital Sign *SD* standard deviation

The Intervention group’s estimated VO2max ranged from 11.2 to 77.3 ml/kg/min with a mean of 41.7 (SD 12.1). Maximum grip strength ranged from 10.9–83.2 Kg with a mean of 35.9 Kg (SD 11.9). Nearly all (97.4%) participants reported some regular exercise activity. The reported types of exercise activity performed at baseline and at follow up is reported in Table 2.

**Table 2:** Type of exercise reported by subjects at baseline and follow-up

	Baseline		3 mo		6 mo		12 mo	
	Control	Intervention	Control	Intervention	Control	Intervention	Control	Intervention
Cardio, N (%)	591 (89.4%)	584 (88.5%)	220 (83.3%)	250 (87.7%)	303 (88.6%)	300 (87.2%)	183 (91.0%)	199 (90.5%)
Sports, N (%)	100 (15.1%)	109 (16.5%)	39 (14.8%)	33 (11.6%)	58 (17%)	52 (15.1%)	33 (16.4%)	31 (13.1%)
Lifestyle activities, N (%)	162 (24.5%)	183 (27.7%)	53 (20.1%)	83 (29.1%)*	64 (18.7%)	86 (25.0%)*	58 (28.9%)	82 (37.3%)
Resistance training, N (%)	174 (26.3%)	192 (29.1%)	83 (31.4%)	122 (42.8%)*	127 (37.1%)	147 (42.7%)	68 (33.8%)	87 (39.5%)
Balance/Flexibility, N (%)	137 (20.7%)	134 (20.3%)	81 (30.7%)	76 (26.7%)	96 (28.1%)	108 (31.4%)	62 (30.8%)	66 (30.0%)
Other, N (%)	42 (6.4%)	40 (6.1%)	26 (9.8%)	34 (11.9%)	44 (12.9%)	36 (10.5%)	17 (8.5%)	16 (7.3%)

*N* number *Indicates *p* < .05 for difference in change from baseline between study groups (Intervention vs. Control)

### Follow-up EVS

During the 1 year follow-up responses were received from 823 subjects (62.6%) with 262 subjects (20%) responding to all 3 contacts. No additional attempts were made to contact participants if they did not respond to our e-mail. Table 3 shows the follow up EVS values. There was no significant change in the EVS from baseline found in either the control or intervention group (*p* = 0.99).

**Table 3:** Change in EVS (min/week) over time

	Baseline EVS	3 mo EVS	6 mo EVS	12 mo EVS
Control				
Mean (SD)	212 (169)	193 (170)	216 (166)	221 (161)
N	656	263	341	200
Intervention				
Mean (SD)	215 (184)	215 (173)	219 (166)	250 (193)
N	659	283	343	219

*N* number of responses, *EVS* Exercise Vital Sign

**Fig. 1:**
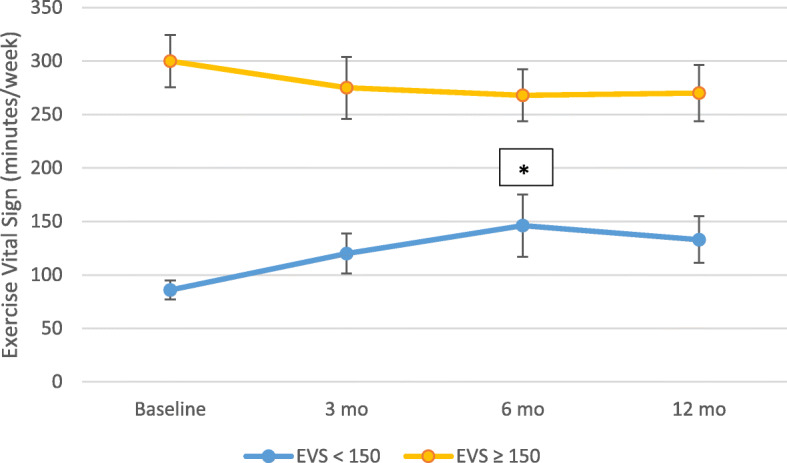
Legend: One year change in Exercise Vital Sign (EVS) in minutes/week for those with baseline below current exercise recommendations of 150 min/week (blue) and those exceeding 150 min/week (orange) in combined control and intervention groups. Error bars indicate 95% Confidence Intervals. (*) signifies *p* < 0.05 relative to baseline

Among the 262 subjects from both groups who provided data at all 3 follow up intervals 82 had a baseline EVS less than the recommended 150 min/week. This group, which includes both control and intervention subjects and a baseline EVS < 150, exhibited an increase in mean EVS over baseline at each follow up point, reaching statistical significance at 6 months (mean EVS 86 (SD = 41) at baseline vs. mean EVS 146 (SD = 135) at 6 months, *p* < 0.05) (Fig. 1). The group meeting or exceeding current recommendations with EVS > 150 at baseline (*N* = 180) showed no significant change.

### Follow-up exercise type

Although the follow up EVS showed no significant differences in the total amount of physical activity, there were significant differences in the type of exercise activity performed (Table 2). At 3 months and 6 months the intervention group exhibited a significant increase in lifestyle activity (29.1% in intervention vs. 20.1% in control at 3 months, 25.0% in intervention vs. 18.7% in control at 6 months, *p* < 0.05 for both 3 months and 6 months). The intervention group also significantly increased resistance exercise at 3 months (42.8% in intervention vs 31.4% in control, *p* < 0.05). This increase in resistance exercise was driven by subjects whose baseline grip strength was less than the norm as they exhibited a significant increase in resistance training throughout the 1 year follow up (32% at baseline vs. 52% at 3 months, 49% at 6 months, and 54% at 12 months, *p* < 0.05). Subjects with grip strength at or above norm significantly increased resistance training at only 6 months (36% at baseline vs. 51% at 6 months, *p* < 0.05) (Table 4).

**Table 4:** Number (%) of subjects reporting resistance training by baseline grip strength norms

	Baseline	3 mo	6 mo	12 mo
Baseline grip strength < norm (*N* = 69)	22 (32%)	36* (52%)	34* (49%)	37* (54%)
Baseline grip strength ≥ norm (*N* = 67)	24 (36%)	31 (46%)	34* (51%)	28 (42%)

*indicates *p* < 0.05 for change from baselin

### Effect of CRF measurements on subsequent EVS

Below norm VO_2max_ (*p* = 1) or grip strength (*p* = 0.75) had no effect on subsequent reported EVS as seen in Table 5. Providing a higher normative value for VO_2max_ also had no effect on subsequent reported EVS (*p* = 0.62).

**Table 5:** Follow up EVS relative to normative data

	Baseline EVS Mean (SD)	3 mo EVS Mean (SD)	6 mo EVS Mean (SD)	12 mo EVS Mean (SD)
Baseline VO2max < norm (*N* = 42)	193 (153)	206 (175)	221 (176)	196 (181)
Baseline VO2max ≥ norm (*N* = 100)	272 (194)	244 (183)	257 (179)	261 (191)
Baseline grip strength < norm (N = 69)	264 (203)	224 (175)	246 (211)	235 (202)
Baseline grip strength ≥ norm (N = 67)	224 (157)	263 (202)	252 (143)	252 (185)

*EVS* Exercise Vital Sign (minutes/week), *N* number of respondents

## Discussion

We did not find that estimates of CRF and MF increased total physical activity over the subsequent year as measured by the EVS. We did find a significant increase in both lifestyle exercise and resistance type exercise in an already very physically active population. Having a below norm grip strength was associated with a significant increase in resistance training throughout the subsequent year. We found that individuals not meeting current recommended physical activity recommendations (EVS < 150 min/week) showed a significant increase in their EVS at 6 months of follow up. This study indicates that recording EVS, providing exercise recommendations and estimating CRF and MF could provide both a useful incentive to stimulate greater interest in exercise, lifestyle physical activity and resistance training. To our knowledge no other study has investigated the effect on subsequent physical activity of VO_2_max estimates or grip strength.

Recognizing the importance of exercise and physical activity to good health, the Surgeon General and others have called for regular assessments of an individual’s physical activity [30, 31]. The National Physical Activity Plan asks healthcare systems to prioritize physical activity assessment, advice, and promotion and regularly assess physical activity as a “vital sign” [32]. The EVS has been advocated as a tool to help accomplish this goal [26]. We used the EVS to quantify the exercise activity in our subjects. It is easily calculated with just two questions and corresponds to current exercise guidelines recommending 150 min of moderately vigorous physical activity per week [27].

Our study population was done in random self-selected volunteers. These volunteers were already very physically active as indicated by the mean EVS of 223.2 with a median value of 180, significantly exceeding current EVS recommendation of 150 [27]. The high level of pre-existing physical activity likely attenuated the impact of the fitness measurements on their future physical activity and limited the utility of the EVS as a measurement tool [33]. We did find, however, that the subjects not meeting current physical activity guidelines did exhibit a significant increase in their reported EVS at 6 months. Both the control and intervention groups exhibited this increase indicating that recording the EVS and providing information on current exercise recommendations likely influenced this change. This observation validates calls for recording exercise as a vital sign. It has previously been reported that systematically recording the EVS during outpatient visits was associated with significant changes in exercise-related clinical counseling and documentation [34]. Consistently meeting physical activity guidelines as measured by the EVS is also strongly associated with a reduced risk for severe COVID-19 outcomes among infected adults [35].

In addition to obtaining the EVS we recorded the types of physical activity both initially and in follow up. We found that the intervention group significantly increased their reported resistance training and lifestyle physical activity relative to controls at 3-months follow-up, despite the much less favorable climate for these activities during winter. The increase in lifestyle activity was sustained at 6 months follow up. A significant increase in resistance training was observed throughout the following year in those individuals with a reported grip strength less than the reported norm. Below norm grip strength appears to stimulate interest in strength training activities in this already very active population. At baseline, 88% of our study population reported participating in some form of cardiovascular exercise but only 28% reported participation in resistance type exercise. This lower level of resistance training is consistent with prior surveys showing only 21.9% of Americans meet muscle strengthening guidelines [34]. This provides greater potential for our assessments and recommendations to have an impact on resistance exercise activity. We did not observe that those subjects with grip strength below the norm increased their EVS even though they did increase their resistance training.

Cardiorespiratory Fitness as measured by VO_2_max is an important indicator of overall health and has significant prognostic implications [12, 14, 15]. Recognizing the significance of CRF the American Heart Association has called for the inclusion of CRF measurement or estimation in routine clinical practice [16]. Despite its importance it is not typically measured in a clinical encounter. This relates to the difficulty of formal VO_2_max measurements. Other forms of estimating VO_2_max such as maximal or sub-maximal treadmill or bicycle exercise testing are also not suited to routine use. VO_2_max can easily be estimated by several formulas based on demographics and reported exercise habits [36, 37]. Estimating CRF from one of these formulas has been associated with CVD and all-cause mortality independent of other risk factors [38]. The estimated CRF from formulas however are significantly influenced by the subjective reporting of exercise activity. We elected to use a step test that had been previously validated in a geriatric population [15]. This test in younger individuals and other populations has been found to be less accurate in the measured VO_2_max yet still felt to be useful in classifying CRF [39]. When this step test has been used to measure CRF to aid in exercise prescription a significant improvement in VO_2_max at 12 months was found compared with baseline measures [40].

We felt that providing a CRF estimate requiring actual physical activity with the step test would more likely influence future exercise behavior than an estimate of CRF from a formula. We did not observe significant changes in the EVS in either the control group or the intervention group over 1 year of follow up. The fitness assessments did not appear to influence this very active population’s physical activity as measured by the EVS. We also did not observe an increase in follow up EVS in those individuals who were reported to have an estimated VO_2_max below the provided norm even when the norm was increased from “good” to “superior”.

We used a hand-grip dynamometer to estimate muscular strength. This test is inexpensive, convenient and previously demonstrated in multiple studies to be a clinically significant marker of sarcopenia and correlate with lower extremity muscle power and mobility (20). In a large longitudinal population study, measurement of grip strength was found to be a simple, inexpensive risk-stratifying method for all-cause death, cardiovascular death, and CVD (21). Grip strength is predictive of mortality in both young adults [41] and middle age [42]. Low grip strength has been documented to correlate with increased disability in the elderly [43], greater risk for hospitalization [44], cognitive decline [45] and nutritional status [46]. We used grip strength to estimate MF and felt that this measurement would contribute to increased physical activity and resistance training. We did observe an increase in resistance training but not total exercise time. The increase in resistance exercise was largely driven by those individuals with grip strength below norm. Given the overall high level of baseline physical activity and lower level of resistance training at baseline it appears that having a below norm grip strength shifted physical activity to resistance training from other activities.

The strengths of this study are its size and diversity. The study participants exhibited a wide range of age (18–92 mean 46) and BMI (16–61, mean 25).

The study limitations were the self-selected population that was predominately white (92.3%) and already very active as exhibited by the high baseline EVS. In this active group the EVS may have been less accurate in measuring their physical activity as the EVS has been shown to under-report physical activity measured by accelerometer and may be best used for identifying individuals not meeting current physical activity guidelines [33]. Other limitations of using the EVS are the absence of a specific time frame and the inability to differentiate exercise intensity. This active group also may have been more receptive to feedback on their CRF and MF accounting for the short term significant increases in the lifestyle and resistance physical activity but with less potential to observe an increase in EVS over time. The results are also limited by the self-reported nature of the data and follow up data being provided from only 62.5% of the study population. We also did not perform follow up CRF or MF measurements.

In summary we did not find that our measurements of CRF and MF using a step test and grip strength increased overall exercise or physical activity as measured by the EVS during the ensuing year. The utility of our intervention was likely limited by a self-selected very active population. Less active individuals in both the control and intervention groups (those not meeting current exercise guidelines) did significantly increase their reported exercise activity at 6 months. We found that the fitness measurements appeared to stimulate an increase in lifestyle and resistance training exercise at short term follow up and that the increase in resistance training was largely driven by those having a below norm grip strength. This indicates a potential benefit of recording the EVS and providing current exercise recommendations to less active individuals. Even very active individuals may benefit from measuring grip strength and providing norms to stimulate greater participation in resistance training activities. Given these encouraging improvements in exercise activity it may be useful to more widely record EVS and perform CRF and MF estimates.

## Conclusions

In a very active population providing VO_2_max estimates and grip strength measurement did not produce an increase in overall physical activity however it did shift activity to increased lifestyle physical activity and resistance training. Recording the EVS and providing exercise recommendations did result in a significant increase in overall physical activity in those individuals not meeting current physical activity recommendations. Wider adoption of the EVS and grip strength measurement could be effective in promoting physical activity and resistance training.

## Data Availability

The datasets used and analyzed during the current study are available from the corresponding author on reasonable request.

## Abbreviations

CVD: Cardiovascular disease
CRF: Cardiorespiratory fitness
VO_2_max: Maximum oxygen uptake
MF: Muscular fitness
EVS: Exercise vital sign
